# Altered deposition of inhaled nanoparticles in subjects with chronic obstructive pulmonary disease

**DOI:** 10.1186/s12890-018-0697-2

**Published:** 2018-08-06

**Authors:** Jonas K F Jakobsson, H Laura Aaltonen, Hanna Nicklasson, Anders Gudmundsson, Jenny Rissler, Per Wollmer, Jakob Löndahl

**Affiliations:** 1 Division of Ergonomics and Aerosol Technology, Lund University, Lund, Sweden; 2 Department of Translational Medicine, Lund University, Malmö, Sweden; 3 Chemistry, Materials and Surfaces, SP Technical Research Institute of Sweden, Lund, Sweden

**Keywords:** Lung deposition, Nanoparticles, Emphysema, Chronic obstructive pulmonary disease, Inhalation exposure, Human, In vivo study

## Abstract

**Background:**

Respiratory tract deposition of airborne particles is a key link to understand their health impact. Experimental data are limited for vulnerable groups such as individuals with respiratory diseases. The aim of this study is to investigate the differences in lung deposition of nanoparticles in the distal lung for healthy subjects and subjects with respiratory disease.

**Methods:**

Lung deposition of nanoparticles (50 and 100 nm) was measured after a 10 s breath-hold for three groups: healthy never-smoking subjects (*n =* 17), asymptomatic (active and former) smokers (*n =* 15) and subjects with chronic obstructive pulmonary disease (*n =* 16). Measurements were made at 1300 mL and 1800 mL volumetric lung depth. Each subject also underwent conventional lung function tests, including post bronchodilator FEV_1_, VC, and diffusing capacity for carbon monoxide, D_L,CO_. Patients with previously diagnosed respiratory disease underwent a CT-scan of the lungs. Particle lung deposition fraction, was compared between the groups and with conventional lung function tests.

**Results:**

We found that the deposition fraction was significantly lower for subjects with emphysema compared to the other subjects (*p* = 0.001–0.01), but no significant differences were found between healthy never-smokers and smokers. Furthermore, the particle deposition correlated with pulmonary function tests, FEV_1%Pred_ (*p* < 0.05), FEV_1_/VC_%Pred_ (*p* < 0.01) and D_L,CO_ (*p* < 0.0005) when all subjects were included. Furthermore, for subjects with emphysema, deposition fraction correlated strongly with D_L,CO_ (Pearson’s *r* = 0.80–0.85, *p* < 0.002) while this correlation was not found within the other groups.

**Conclusions:**

Lower deposition fraction was observed for emphysematous subjects and this can be explained by enlarged distal airspaces in the lungs. As expected, deposition increases for smaller particles and deeper inhalation. The observed results have implications for exposure assessment of air pollution and dosimetry of aerosol-based drug delivery of nanoparticles.

## Background

Factors determining the deposited dose of ambient air pollution include the properties of the inhaled particles as well as individual anatomical and physiological characteristics, such as lung morphology and breathing pattern. The objective of this study was to investigate respiratory tract deposition of nanoparticles in the distal lung in healthy never-smoking subjects, asymptomatic smokers and subjects with diagnosed chronic obstructive pulmonary disease (COPD).

Numerous epidemiological studies show links between airborne pollutants and adverse health effects in terms of both increased morbidity and mortality [1]. By number, ultrafine particles normally dominate in both indoor and outdoor air, and are important for human health, as they are known to be able to penetrate to the deep lungs and deposit there with high efficiency. The material in soluble particles easily translocate into other parts of the body, while insoluble particles are believed to accumulate in the lungs because of low clearance rates in the alveolar region [2]; both prospects may have adverse effects on human health.

Established lung models and previous experimental studies agree that nanoparticles (< 100 nm) to a high degree deposit in the distal lung, with the exception of the very smallest particles below 5–10 nm that deposit higher up in the respiratory tract due to their high diffusion velocity. However, vast intersubject variability have been observed in experimental studies [3–6]. Intersubject variability has also been investigated by modelling [7–9], although there is generally a substantial lack of theoretical calculations for deposition of particles in unhealthy lungs. Experimental data of respiratory tract deposition of nanoparticles is limited to about 50 studies [10], most containing data for small groups (< 10 subjects). Because of differences in methodology and low numbers of subjects, it is not trivial to compare the results between studies or with theoretical models. Especially studies using controlled breathing patterns for larger groups and for subjects with respiratory disease are scarce.

To the best of our knowledge, only five studies have previously reported lung deposition data for nanoparticles in subjects with obstructive lung disease [2, 11–14], and of these only two present data for subjects with explicitly defined emphysema [12, 14]. In a recent publication [14], which included a subset of the material in this study, we have shown that deposition of nanoparticles is reduced in subjects with COPD compared to never-smokers and correlates with the degree of emphysema. This paper extends the observations to a larger number of subjects, two different particle sizes and two different volumetric sample depths. Anderson et al. [11] measured lung deposition for particles in the size range 40–240 nm in obstructive, restrictive and healthy subjects, and reported increased particle deposition efficiency, for subjects with airflow obstruction. Möller et al. [2] also reported increased particle deposition efficiency for obstructive subjects and smokers compared to healthy non-smoking subjects. The measurements were performed with aerosol boluses aimed at the peripheral lung (800 mL volumetric lung depth) and to the more central regions (150 mL volumetric lung depth). Increased deposition efficiency was reported for the peripheral lung, but not for the central airways. Brown et al. [12] showed increased particle deposition for obstructive subjects with bronchitis and decreased particle deposition for emphysematous subjects, compared to healthy subjects. Similar results were also presented by Löndahl et al. [13] who reported decreased particle deposition for COPD subjects for particles < 100 nm and slightly increased lung deposition for larger particles. Thus, depending on phenotype of COPD, the disease can both increase and decrease the deposition of inhaled aerosols.

For particles in the diffusion-dominated range (< 300 nm), the main experimental parameters that govern deposition efficiency are particle size, residence time in the peripheral lung and distance to available surfaces. Previous studies reporting data from experiments using spontaneous [4, 6, 13, 15–17] or controlled [11, 18] natural breathing patterns determine deposited dose of airborne nanoparticles under real life exposure conditions and for different groups, but generally do not provide information on regional deposition.

In this study, we report data for deposition of monodisperse nanoparticles in the peripheral airways (> 1300 mL volumetric sample depth) for three groups of subjects. In order to isolate effects from differences in lung structure, we performed the experiments with a controlled breathing pattern including a breath-hold. We hypothesised that the deposition of nanoparticles will differ between healthy subjects, and subjects with diagnosed respiratory disease. Respiratory disease was assessed with pulmonary function testing and, for subjects with previously diagnosed disease, computed tomography (CT). A detailed analysis of the CT results is provided by Aaltonen et al. in a separate publication [14]. Smoking is known to affect the lungs even for individuals with relatively short smoking history [19, 20] and therefore smokers without symptoms of respiratory disease were included as a separate group.

## Methods

### Subjects and experimental design

The study was performed on COPD-patients recruited from the department of pulmonary medicine at Skåne University Hospital in Malmö, Sweden, and at a local primary health care clinic, and on two groups of subjects with no symptoms of respiratory disease, whereof one was never-smokers and the other active or former smokers.

All subjects underwent conventional lung function tests, according to current recommendations for diagnosis of respiratory disease [21–23]. Most of the patients (*n =* 15/16) with previously diagnosed COPD underwent a CT-scan of the lungs, but the other groups were omitted from this part of the study to reduce the exposure to radiation. In addition to conventional lung function tests, all subjects experienced a total of nine measurements of lung deposition of nanoparticles (three replicates at three different measurement conditions) during a total experimental time of approximately 30 min. The deposition measurements were generally performed directly in connection with the clinical lung function tests, or within 6 weeks.

Division of the subjects into the three groups was made based on lung function tests, smoking history and previously diagnosed respiratory disease (Table 1). The healthy never-smoking group (*n* = 17) was defined as individuals with no previous smoking history and no respiratory disease, normal spirometry (as defined by the Global Initiative for Chronic Obstructive Lung Disease, GOLD [24]), and D_L,CO_. The “asymptomatic smoker” group (*n* = 15) was defined as present or former smokers, with normal spirometry and D_L,CO_, and no known respiratory disease. In this group the subjects had an average estimated smoking history of 14 pack-years (range 3–41). Two subjects were active smokers and reliable data was missing for 3 subjects. The COPD group (*n* = 16) consisted of subjects with diagnosed COPD, with airflow obstruction corresponding to spirometric GOLD-stages 1–4 [24]. In the COPD-group, 3 subjects underwent triple therapy, inhaled corticosteroids (ICS), long-acting beta-agonist (LABA) and long-acting muscarinic antagonist (LAMA), while 2 underwent therapy with ICS and LAMA or LABA, 3 subjects had LABA and LAMA therapy. Three individuals reported no inhaled therapy and data was missing for 5 individuals. In this group 4 subjects were never-smokers with alpha-1 antitrypsin deficiency, 10 subjects ex-smokers and 2 subjects active smokers. The COPD subjects that reported smoking history had an average of 37 pack-years (range 0.5–68). Reliable data for estimating pack-years was missing for 4 of these subjects. There were no significant differences on a group level between the subjects with regards to gender, age, weight, height and VC.

**Table 1:** Subject demographics and lung function

	Healthy Never-Smokers (*n =* 17)	Asymptomatic Smokers (*n =* 15)	COPD (*n =* 16)
Male/Female	9/8	6/9	7/9
Age [y]	64 ± 6	62 ± 3	67 ± 7
Weight [kg]	76 ± 14	76 ± 12	72 ± 13
Height [cm]	173 ± 9	169 ± 9	170 ± 9
FEV_1_%pred	110 ± 15	109 ± 13	61 ± 23^***^
VC %pred	109 ± 20	112 ± 13	107 ± 18
FEV_1_/VC %pred	78.6 ± 4.0	78.4 ± 5.6	44.9 ± 14.2^***^
D_L,CO_ %pred	92.0 ± 10	88.2 ± 13	60.2 ± 22^***†^
GOLD-stage (1/2/3/4)			3/7/5/1

**†** Data for D_L,CO_ for one subject in the COPD group is missing

***Significance < 0.001 level

*Descriptive demographic data (average ± 1 standard deviation) for the subjects, lung function tests performed post bronchodilation, 1.5 mg terbutaline (Bricanyl Turbuhaler, Astra Zeneca, Mölndal, Sweden)*

In total 69 subjects participated, whereof 48 subjects completed the study. Criteria for acceptable quality of particle deposition measurements were measurements with inhalation to > 70% VC, and a complete breathing manoeuvre in less than 17 s. Of the 21 subjects that were omitted, two subjects were not able to exhale the full 1800 mL breath sample, 10 were not able to perform the breathing manoeuvre within the stipulated time, 9 failed to inhale a sufficient volume of test aerosol. It was primarily subjects with more severe COPD that failed to follow the quality criteria, partly due to flow resistance in the inhalation apparatus.

The study was approved by the regional ethical review board of Lund and was performed in accordance with the Declaration of Helsinki. An informed written consent was obtained from all subjects.

### Pulmonary function testing

Each subject underwent conventional lung function tests, according to current recommendations for diagnosis of respiratory disease [21–23], including measurement of forced expiratory volume in one second (FEV_1_) and vital capacity (VC), as well as diffusing capacity for carbon monoxide (D_L,CO_), (Jaeger MasterScreen PFT, IntraMedic, Sollentuna, Sweden). The lung function tests were performed post bronchodilation (1.5 mg terbutaline (Bricanyl Turbuhaler, Astra Zeneca, Mölndal, Sweden). Lung function variables are, if not explicitly stated otherwise, presented as percentage of predicted values, [21, 25].

### Computed tomography

All but one of the patients with previously diagnosed COPD underwent a CT-scan of the lungs. The exclusion of one patient was merely for logistical reasons. A detailed analysis of the CT results, including CT densitometry, is described for a subset of the material in a separate publication [14]. The scans were performed using a multidetector-row CT scanner (Siemens Somatom Definition Flash; Siemens Healthcare, Forchheim, Germany) in suspended full inspiration with a reduced radiation dose, generating an exposure of 120 kV / 15 mAs. The CT scans were visually assessed by a radiologist with 9 years of experience and a radiology resident with 2 years of experience, and classified as having none, mild, moderate or severe emphysema [26].

### Particle deposition measurements

**Fig. 1:**
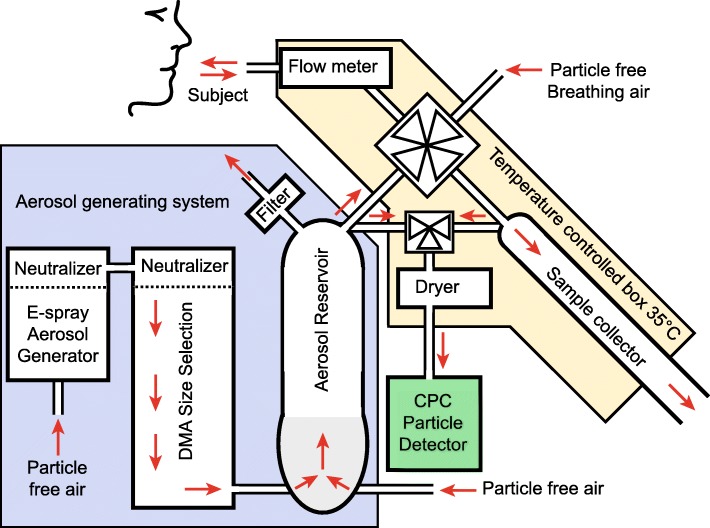
The aerosol deposition measurement system. The aerosol deposition measurement system can be divided into three sub-systems. Aerosol generation system (blue): the test aerosol is generated by an electrospray aerosol, followed by size-selection and dilution with particle free air. Inhalation system (red): a four-way, high-speed, computer-controlled valve directs particle free air or test aerosol to the subject, monitors the breathing pattern and collects exhaled samples from a well-defined volumetric sample depth. The system is enclosed in a temperature controlled box held at 35°c. Particle detection system (green): the particle number concentration in the test aerosol and in exhaled samples are measured with a condensation particle counter

The instrumentation used for the particle deposition measurements has been described in detail elsewhere [27]. An overview is shown in Fig. 1. In short, a test aerosol was produced by aerosolizing polystyrene latex nanospheres (PSL) (Polymer Microsphere Suspension, Microgenics Corp, Fremont CA, US) with an electrospray aerosol generator (Model 3480, TSI Inc., Shoreview, MN, US). The produced aerosol was size controlled by a differential mobility analyser (DMA, Model 3071, TSI GmbH, Aachen, Germany) and diluted with particle free air to a concentration of 3000–6000 particles cm^− 3^. The test aerosol was continuously produced and led into a semi-flexible aerosol reservoir with an exhaust in a flow-through design that ensured a steady supply of fresh test aerosol during the measurements. A high-speed computer controlled valve administered the test aerosol or particle-free air to the subjects through a mouthpiece connected to a pneumotachograph flow meter (as used in MasterScreen PFT, Viasys GmbH - Erich Jaeger, Hoechberg, Germany). The high speed valve was also connected to a sample collector, enabling sampling from well-defined exhaled volumes corresponding to specific volumetric lung depths. The particle concentration was monitored in the aerosol reservoir, and in the exhaled samples by a condensation particle counter (CPC, Model a20, Airmodus Ltd., Helsinki, Finland). The instrument was constructed according to the design criteria described by Löndahl et al. [10] and particle losses were characterized and compensated for [27].

**Fig. 2:**
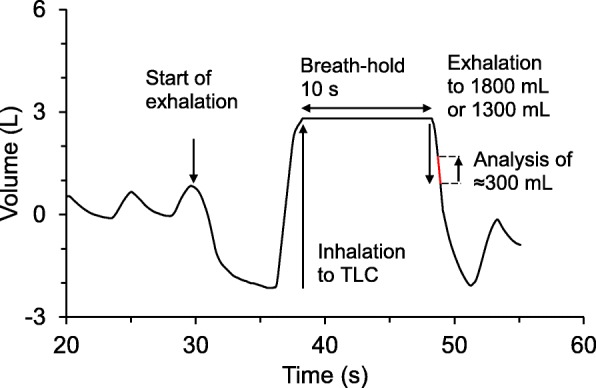
The breathing pattern. The breathing protocol, illustrated by breathing data for a standard measurement, recorded at 100 Hz. The protocol starts with a period of tidal breathing of particle free air. The breathing manoeuvre is initiated with exhalation to RV followed by an inhalation of the test aerosol to TLC, a breath-hold of 10 s and then relaxed exhalation. A sample is collected from the exhaled air after a controlled volume has passed the sample collector. The particle number concentration is monitored continuously in the test aerosol until the inhalation, and then determined in the exhaled sample immediately after exhalation. The aerosol residence time in the lungs is calculated from the recorded breathing pattern

The deposition measurements (Fig. 2) started with a period when the subject breathed particle free air to wash out resident ambient air particles and to get accustomed to the instrument. After a minimum period of 30 s, the subject performed a total exhalation to residual volume (RV), followed by a deep inhalation to total lung capacity (TLC). When the inhalation started, the altered flow direction detected by the pneumotachograph automatically triggered the high speed valve to switch, so that the subject inhaled the test aerosol. When the subject reached TLC all valves were shut, and the subject held his breath for a determined time period (10 s). Thereafter the valve opened towards the sample collector and the subject exhaled a predetermined volume, before the valve was turned and the rest of the breath was discarded to exhaust. Measurements were carried out with spherical 50 nm particles (mobility equivalent diameter) and from exhaled samples corresponding to air with a sample front at 1300 mL and 1800 mL volumetric lung depth, and with 100 nm particles from 1800 mL volumetric lung depth. The breathing pattern is shown in Fig. 2.

The particle deposition fraction (DF) is defined as the fraction of the exhaled particle concentration related to the inhaled particle concentration, and is corrected for particle losses in the instrument according to the relation:

$$ DF=1-\frac{C}{C_0{R}_i\left({t}_i\right)} $$

Here C is the particle number concentration in the exhaled sample and *C*_0_ is the particle number concentration in the inhaled test aerosol. *R*_i_ is the instrument recovery, which describes particle losses in the instrument as a function of residence time during measurements, *t*_i_. The relation for *R*_i_ is semi-empirical and derived from theory for particle diffusion in confined spaces combined with data for simulated breathing manoeuvres with a physical model, as described in detail elsewhere [27]. The measured DF was reported as the mean value of three measurements. The standard deviation for the measurements was typically 0.001, 0.007 and 0.02 for 50 nm particles/1300 mL, 50 nm particles/1800 mL and 100 nm/1800 mL sample depth respectively.

For the breathing pattern used, the respiratory flow-rates during inhalation and exhalation has previously been shown to have minimal influence on the measurements [27], as long as the total residence time in the lungs does not change. Thus the subjects could inhale and exhale at uncontrolled breathing flow-rates. The residence time in the lungs was defined as the time between midpoint times of inhalation and the exhaled sample in similarity with standard for measurement of D_L,CO_ [22].

### Analysis

Although all subjects used similar breath-hold times, there were variations in the total residence time of the particles in the lungs due to differences in exhaled flow rate. To decrease variations in measured DF that were only due to these differences in breathing pattern, we time normalized data to 13 s total residence time. The time normalization was based on the previously observed relationship between DF and breath-hold time for 50 nm and 100 nm particles in healthy subjects [27], which was also found in the present group. We used a linear approximation of the exponential diffusion deposition equation [28] to avoid overestimates of the time correction. Even though the correction may be less valid for severely diseased subjects, this normalization was considered necessary to avoid bias due to the prolonged aerosol residence time in the lungs caused by slower exhalation by the COPD subjects. In the previous study by Anderson et al. [11], a deviation in breathing pattern among the COPD subjects biased the data and made valid conclusions more difficult. The linear approximation was also implemented with the aim to be a conservative approach. The effect of the time normalization on the DF was on average a decrease of 0.015 ± 0.010 for 50 nm particles and 1300 mL sample depth, 0.012 ± 0.008 for 50 nm particles and 1800 mL sample depth, and 0.030 ± 0.020 for 100 nm particles and 1800 mL volumetric sample depth. Statistical analysis was made with IBM SPSS Statistics 23 (Release 23.0.0.0, 2015). Non-parametric statistics, (Kruskal-Wallis test followed by Mann-Whitney) was used for group comparisons, and Pearson’s chi square analysis was performed to check for gender differences between groups. The correlation analysis was calculated with Pearson’s product moments. The significances considered were those at the 0.05 or higher levels. For group comparisons between three groups, Bonferroni correction was applied, thus the significance level was adjusted to 0.05/3 = 0.016.

## Results

The average demographics and measured lung function for the included subjects are shown in Table 1. Using the Kruskal-Wallis test, it was shown that there were no significant differences on a group level between the subjects with regards to age, weight, height and VC. Pearson’s chi square analysis revealed no significant gender differences between the groups.

**Fig. 3:**
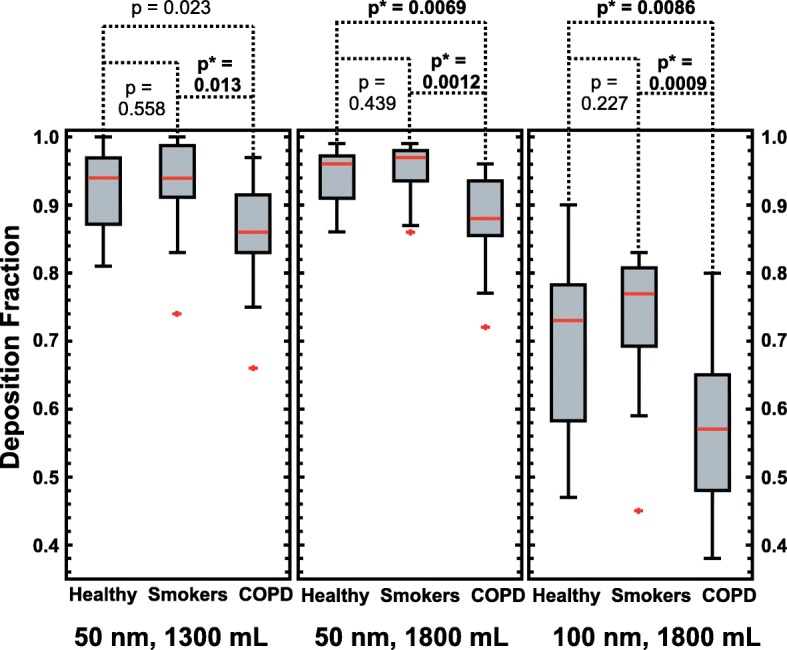
Boxplot of particle deposition for the three groups. Particle deposition fraction, DF, in the peripheral lungs for the three groups, measured for 50 nm particles and from 1300 mL volumetric sample depth (left panel), 50 nm particles and 1800 mL sample depth (middle panel) and 100 nm particles and 1800 mL sample depth (right panel). The red line indicates the median, the box 25th and 75th percentiles and the whiskers minimum and maximum values (two outliers displayed as a red marks). The *p*-values (Mann-Witney asymptotic significance, 2-tailed) are given in the figure. Significant differences between the groups are indicated with *. The 5% significance level for rejecting the null hypothesis is adjusted to 0.016 (Bonferroni correction)

The average deposition fractions, DF, at different sample volumes and particle sizes are shown in Fig. 3 and the mean values for each group are provided in Table 2.

**Table 2:** Particle deposition fractions

	DF 50 nm 1300 mL	DF 50 nm 1800 mL	DF 100 nm 1300 mL
Healthy Never-Smokers (*n =* 17)	0.92 ± 0.06	0.94 ± 0.05	0.69 ± 0.12
Asymptomatic Smokers (*n =* 15)	0.93 ± 0.08	0.95 ± 0.04	0.73 ± 0.11
COPD (*n =* 16)	0.86 ± 0.08^*^	0.88 ± 0.07^*^	0.57 ± 0.12^*^
All Subjects (*n =* 48)	0.90 ± 0.08	0.92 ± 0.06	0.66 ± 0.14

* Significance < 0.016 level. Particle deposition fraction, DF, for the different groups (average DF ± 1 standard deviation). Significant differences compared to the healthy reference group marked by *, (Bonferroni adjusted significance level 0.016)

The DF was significantly higher for the healthy never-smoking subjects and the asymptomatic smokers, than for the subjects diagnosed with COPD. There were no significant differences in DF between the healthy never-smokers and asymptomatic smokers and no significant gender differences. Neither did DF correlate with age.

**Fig. 4:**
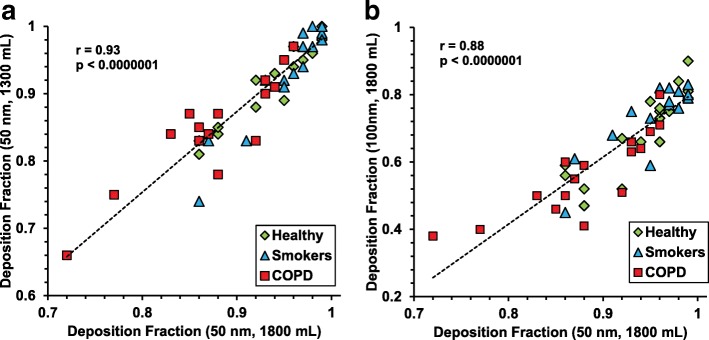
(**a**, **b**). Correlations for volumetric sample depth and particle size. The relation between lung particle deposition measured at different sample depths and for different particle sizes. In **a** (left panel), for 50 nm particles at sample depths 1300 ml and 1800 ml, and in **b** (right panel), for 50 nm and 100 nm particles at sample depth 1800 ml. Pearson’s correlation coefficient and *p*-values are indicated in the figure. Healthy = green, smokers = blue, COPD = red

Figure 4 shows the correlation between the DF measured for each individual for different particle sizes and volumetric sample depths. DF varied in a consistent way for the subjects – with some subjects having a low DF for all measurements and others a high. Especially in the COPD group there was a larger intra-subject variability in DF (coefficient of variance 8–21% for the COPD group compared to 5–16% on average for the healthy groups), and this group also included subjects with a considerably low DF.

**Fig. 5:**
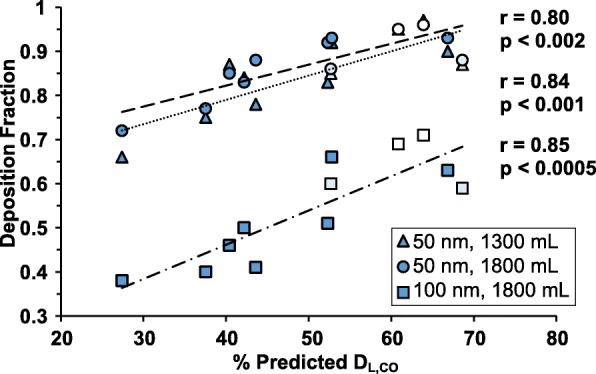
Correlation between particle DF and D_L,CO_ for COPD subjects. Correlation between D_L,CO_ and particle deposition for emphysematous subjects (*n =* 12). The included subjects were confirmed to have emphysema by visual CT evaluation and graded to have mild (*n =* 2), moderate (*n =* 2) and severe (*n =* 8) emphysema. The visual grading is indicated by colour intensity (white = mild, light blue = moderate, dark blue = severe). Four subjects were omitted from the analysis based on a D_L,CO_ > 95% of predicted, or missing data. Of these one had no emphysema and one was graded as having mild emphysema but 97% of predicted D_L,CO_, CT data was missing for one subject and D_L,CO_ was missing for one subject

The measured DF was found to correlate with FEV_1%pred_ and FEV_1_/VC_%pred_ when all (*n =* 48) subjects were included in the analysis. This is most likely an effect of group differences (Table 3). There was no significant correlation between DF and sex, age, weight, height or VC for any of the groups or when all subjects were analysed together. For healthy never-smokers a negative correlation was found between DF and FEV_1%pred_. The correlation was weak for 50 nm particles, at 1300 mL and 1800 mL respectively (Pearson’s *r* = − 0.489, *p* = 0.047 and Pearson’s *r* = − 0.491, *p* = 0.045), but much stronger for 100 nm particles at 1800 mL sample depth (Pearson’s *r* = − 0.728, *p* = 0.001). No significant correlation was found between the spirometry parameters and DF for the COPD group or for the asymptomatic smokers.

**Table 3:** Correlation between deposition fractions and lung function

		DF	DF	DF
		50 nm 1300 mL	50 nm 1800 mL	100 nm 1800 mL
FEV_1%pred_	Pearson’s r	0.317^*^	0.391^**^	0.357^**^
	p-value	0.028	0.0060	0.013
FEV_1_/VC_%pred_	Pearson’s r	0.452^**^	0.530^***^	0.574^***^
	*p*-value	0.0013	0.00011	0.000020
DL_,CO %pred_^†^	Pearson’s r	0.494^***^	0.571^***^	0.518^***^
	*p*-value	0.00049	0.000034	0.00023

**†** *n =* 46, D_L,CO_ data for two subjects is missing

*Significance < 0.05 level

**Significance < 0.01 level

***Significance < 0.001 level

*Pearson’s correlation coefficients, r, and p-values for the associations between particle deposition and clinical lung function tests for all subjects (n = 48)*

Significant correlation between DF and D_L,CO %pred_ was found for the COPD group. Especially when studying the subjects with < 90% D_L,CO %pred_, (Fig. 5). Computed tomography data was available for all but one of these subjects, and a visual grading [26] of the group confirmed that the subjects with > 90% D_L,CO %pred_ had no or minor emphysema, while the other subjects generally had moderate to severe emphysema. An analysis of the CT results, including CT densitometry, is described in a separate publication [14]. The correlations for all subjects are summarized in Table 3 and the correlation between D_L,CO_ and DF for 50 nm particles (1300 mL, 1800 mL sample depth) and 100 nm (1800 mL sample depth) for the COPD group with < 90% D_L,CO %pred_ is shown in Fig. 5. There was no correlation between DF and D_L,CO_ for the healthy subjects.

## Discussion

This study presents measurements of deposition of nanoparticles in the distal lungs, for healthy never-smoking subjects, asymptomatic (active or former) smokers and subjects with diagnosed COPD. Measurements were performed using a controlled breathing pattern including a breath hold. There was a significant difference in particle deposition between COPD patients and the other subjects, but no significant differences between the healthy never-smokers and asymptomatic smokers. Significant correlation was also found between DF and D_L,CO%pred_ for patients with confirmed emphysema, but not for the other subjects.

The method used in this study is specifically designed to examine particle deposition in the alveolar region of the lung during a breath-hold. Although the controlled breathing pattern deviates from normal tidal breathing, and therefore does not reflect a typical everyday exposure scenario, it enables investigation of specific relations between lung morphology and particle deposition mechanisms. We used monodisperse nanoparticles, known to deposit mainly by diffusion in the distal lung, with small influence of the larger conducting airways, [28]. Time normalization was applied to minimize differences in breathing pattern, and measurements were performed at full lung inflation. As a consequence, most of the observed particle deposition is expected to occur by diffusion during the breath-hold, with the lung at a fixed state of inflation. It is therefore likely that the variation of particle deposition in this study primarily reflects individual variation of lung morphology in the peripheral lung.

Mainly two features of lung morphology can be assumed to affect lung deposition in diseased lungs: narrowing of the bronchioles (bronchiolitis), causing increased deposition, and enlargement of distal airspaces (emphysema) with the opposite effect [29]. Generally it can be supposed that COPD subjects have varying proportions of both bronchiolitis and emphysema, and that the asymptomatic smokers primarily have some irritation and narrowing of the bronchioles [19, 20, 30].

The observed decrease in DF for the COPD subjects in this study is most probably caused by the presence of pulmonary emphysema, a conclusion that is supported by the correlation between DF and D_L,CO_ for the COPD group. However, other lung features that could provide an alternative explanation should be further investigated. Local alterations to the lung morphology, such as chokes, collapsing airways or poorly ventilated airspaces (i.e. bullae) most probably also have some effect on particle deposition. However, these conditions would more likely increase than decrease particle deposition, and thus do not explain the observed findings. It is also important to note that even though we found a significantly lower DF for subjects with COPD in this study, the total particle burden may be increased for this group during natural tidal breathing, as individuals with COPD generally have increased minute ventilation and thus process more air than healthy individuals [13, 31].

Previous studies of lung deposition of nanoparticles (< 100 nm) are scarce and the reported data show much divergence. This can be attributed to differences in methodology as well as differences between individual subjects [5]. Several studies of the deposition of nanoparticles have been performed with natural or spontaneous breathing patterns [4, 12, 13, 15–17, 32–38]. Generally, the studies report lower particle deposition than found in the present study, as expected since most of these previous studies are performed at lower degrees of lung inflation and shorter residence times of aerosol in the lung. Studies with controlled breathing patterns [2, 11, 12, 39–43], usually defined by controlled volumes and flow rates, result in particle deposition being measured at different degrees of lung inflation and from different anatomical regions of the lung. Several studies report increased DF both for smokers [2, 17] and for subjects with airflow obstruction [16, 37]. This is contrary to our results. However, most of the studies measure particle deposition at shallower volumetric lung depths, leading to a stronger influence of the deposition in the conducting airways (such as turbulent diffusion at local anatomical alterations) rather than in the alveolar region of the lung targeted by this study.

Several mechanisms for the increased DF of nanoparticles in obstructed subjects have been proposed by Anderson et al. [11]. The obstructed subjects in their study (*n =* 5) were characterized as having mainly chronic bronchitis or asthma. Among the suggested mechanisms, it was shown that failure to comply with the predetermined breathing pattern, resulting in longer residence times for the patients, did not fully explain the observed differences in deposition, and they suggest that abnormal expiratory collapse of airways and narrowing of airway calibre combined with excessive airway secretion is a more likely mechanism.

Using a similar breathing protocol and particle size (92–103 nm) as the present study, Möller et al. [2], found significant differences in DF between healthy subjects (*n =* 9), asymptomatic smokers (*n =* 10) and COPD patients (*n =* 7), with increased DF for smokers and COPD patients. The particle deposition was measured after bolus administration of aerosol to 800 and 150 mL volumetric lung depth. Differences were only found at 800 mL. The authors concluded that the observed increased DF was caused by narrowing of the distal airways, bronchiolitis, and that at the more shallow bolus, (reaching mainly central airways), the relative changes in airway diameter for obstructive subjects were not expected to have a strong influence on diffusional particle deposition.

To our knowledge, the only study reporting data for confirmed emphysematous subjects (with exception for the measurements performed within this project [14]), Brown et al. [12], agrees with our results [14]. In that study lower DF is reported for subjects with emphysema (*n =* 3) than for healthy subjects (*n =* 9), and higher DF for subjects with bronchitis (*n =* 7) than for healthy subjects. Another study by Löndahl et al. [13], using continuous breathing of polydisperse diesel exhaust particles has also showed lowered DF in subjects with COPD in the size range < 100 nm, similar to the results in this study, but increased DF for larger particles, that have a higher probability to deposit by inertial impaction and sedimentation in the conducting airways.

More data on DF in the peripheral lungs are available for larger aerosol particles, than for nanoparticles. Techniques have been developed to use airborne particles to infer information about the lung, using controlled breathing patterns similar to the present study, but with particles close to 1 μm in size [44, 45]. In similarity with our results, these studies also found a decreased DF in the distal lung for subjects with emphysema [46–50]. Particles around 1 μm inhaled at low flow rates are assumed to deposit mainly by sedimentation, and models have been developed to use these techniques to derive effective airway diameters (EADs) from particle deposition measurements [51–53]. The most well-explored of these techniques are the different versions of the aerosol-derived airway morphometry technique (ADAM) [45] and the bolus dispersion technique [44]. ADAM has been proposed as a diagnostic method with specificity for pulmonary emphysema [45] although the technique has not been established in clinical practice.

The conclusion that the decreased DF observed in the COPD group in this study is an effect of enlarged distal airspaces is also consistent with the correlations between lung function tests and DF. The influence of particle deposition in the upper airways have been minimized by the choice of particle size, volumetric sample depth and the inclusion of a breath hold. Correlations were found both with spirometric parameters and D_L,CO_ when all subjects (*n =* 48) were included in the analysis, but not within all of the separate groups. Interestingly, correlation with D_L,CO_ was only found within the COPD group, and a negative correlation to spirometry was only found in the healthy never-smoking group. The reason for the negative correlation between FEV_1_ and particle deposition found for the healthy never-smoking subjects is not clear, but may be an effect of flow rate-dependent particle losses during the dynamic part of the breathing manoeuvre. The effect may be present for the other groups, but distorted by less uniform changes to the conducting airways and therefore not detected in this study. The found correlations agree with the results presented by Brown et al. [12], who report significant correlation between both D_L,CO_ and particle deposition for the emphysematous subjects but not for the non-emphysematous subjects, who instead had a weak (*p* = 0.045) correlation between particle deposition and spirometry.

The DF was consistently higher for 1800 mL than for 1300 mL volumetric sample depth in all groups, but the effect of volume is small compared to the influence of particle size. The observed relation between DF and volume was not merely an effect of different residence times in the lungs and in agreement with previous studies [2, 11], which attribute the increased DF to the increasingly narrowing dimensions in the tracheobronchial tree. The lower DF for 100 nm particles, compared to 50 nm particles, is consistent with the theory of Brownian diffusion and previous studies [4, 11, 15, 16, 43, 54].

D_L,CO_ is known to correlate with degree of emphysema, and thus the correlation between D_L,CO_ and DF suggests that the lower DF in the diseased group is not primarily caused by airflow obstruction, but rather by the extent of emphysema. Visual grading of the available CT-data for the obstructed subjects confirmed that those with D_L,CO_ < 90% of the predicted values had varying degrees of emphysema. This is also consistent with the full data set. As most subjects from the healthy groups are expected to have no or neglible emphysema, no correlation was expected for the healthy subjects. Correlations between particle deposition and CT-densitometry [14] also support this interpretation. Similar relations between particle deposition and lung function tests have also been observed for larger particles [46, 55, 56].

Although the number of subjects with COPD, to the best of our knowledge, exceeds previous comparable studies [10], the group is limited and more data is needed to support the conclusions. No clear differences were detected between healthy never-smokers and asymptomatic smokers. This could likely be explained by the classification of smokers (any reported tobacco use qualified the subject as a “smoker”) that resulted in a diverse group with a wide range of smoking history. A more targeted recruitment of a group of “heavy smokers” may have generated clearer results. Another limitation that should be investigated further is the ability to perform measurements on diseased subjects. Several subjects were excluded from the study for not meeting the quality criteria of the particle deposition measurement. It was also observed that the intra-subject variability was elevated for the COPD group. The variability may reflect individual lung abnormalities, but may also be related to the ability of COPD subjects to comply with the measurement protocol. A re-design of the breathing manoeuvre with a shorter breath-hold and smaller exhaled volumes may be considered for future studies.

The results presented in this study suggest that measurement of lung deposition of nanoparticles could be utilized to detect and quantify respiratory disease, potentially giving medically relevant information about diffusion distances in the lungs similar to magnetic resonance imaging (MRI) with hyperpolarized noble gas [57, 58]. Such a technique, named AiDA, has recently been suggested by Löndahl et al. [29]. By measuring particle deposition for different breath-holding times and determining the particle half-life time in the lungs, it is possible to determine effective airspace radii in the distal lung. In two recent proof-of-concept studies the technique was compared to MRI [57] and CT [14]. In the MRI study the authors found that AiDA-derived airspace radii correlated with lung tissue density. In the CT study it was found that particle deposition correlated with CT densitometry for emphysematous subjects. These findings are in agreement with the results of this study.

## Conclusions

This study presents measurements of nanoparticle deposition in the distal lung for healthy subjects and subjects with COPD during a breath-hold. DF was significantly lower for subjects with COPD compared to the healthy group, which can be explained by enlarged distal airspaces caused by emphysema. The conclusion was further strengthened by a strong correlation between DF and emphysema, as assessed by D_L,CO%pred_, within the COPD group. The results show that lung morphology has a significant influence on the deposition of nanoparticles in humans, and should be considered when estimating the lung burden of airborne particles, when identifying especially vulnerable groups and in dosimetry of aerosol based drug delivery.

## Abbreviations

ADAM: Aerosol-derived Airway Morphometry
AiDA: Airspace Dimension Assessment by nanoparticles
bCPC: (Butanol) Condensation particle counter
COPD: Chronic obstructive pulmonary disease
CT: Computed tomography
DF: Deposition fraction
D_L,CO_: Diffusing capacity for carbon monoxide
DMA: Differential mobility analyser
FEV_1_: Forced expiratory flow during one second
GOLD: Global Initiative on Obstructive Disease
ICS: Inhaled corticosteroid
LABA: long-acting beta-agonist
LAMA: long-acting muscarinic antagonist
PSL: Polystyrene Latex
RV: Residual volume
TLC: Total lung capacity
VC: Vital capacity
